# Supplementary figures and images for: Dilated cardiomyopathy-associated RNA-binding motif protein 20 regulates long pre-mRNAs in neurons
Source: eLife. 2026 Jan 12;14:RP104808. doi: 10.7554/eLife.104808 (PMC12795506; doi:10.7554/eLife.104808)

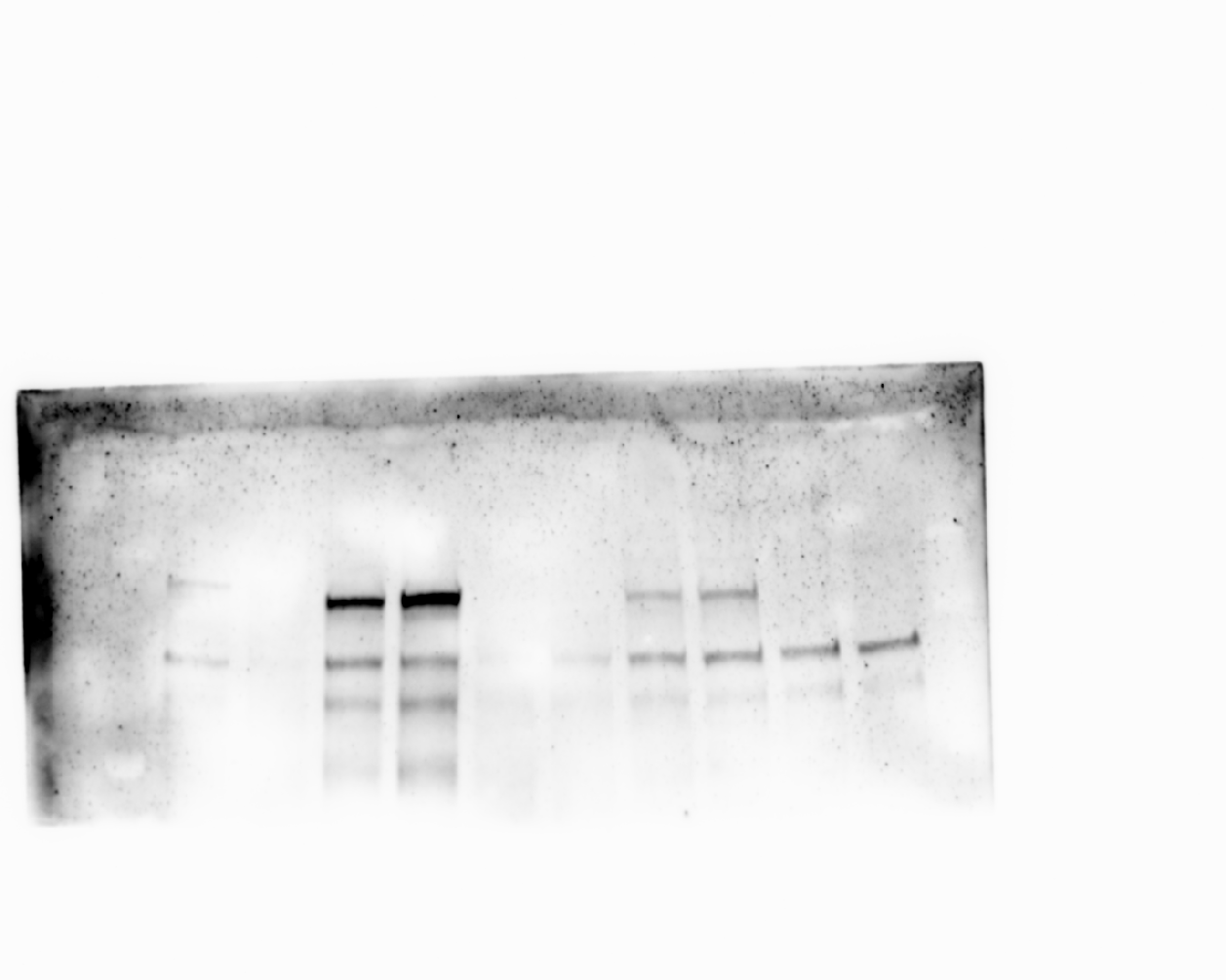

Supplement: Figure 1—source data 1. [file elife-104808-fig1-data1.zip › Raul 2025-01-09 13h05m04s(Chemiluminescence).tif]

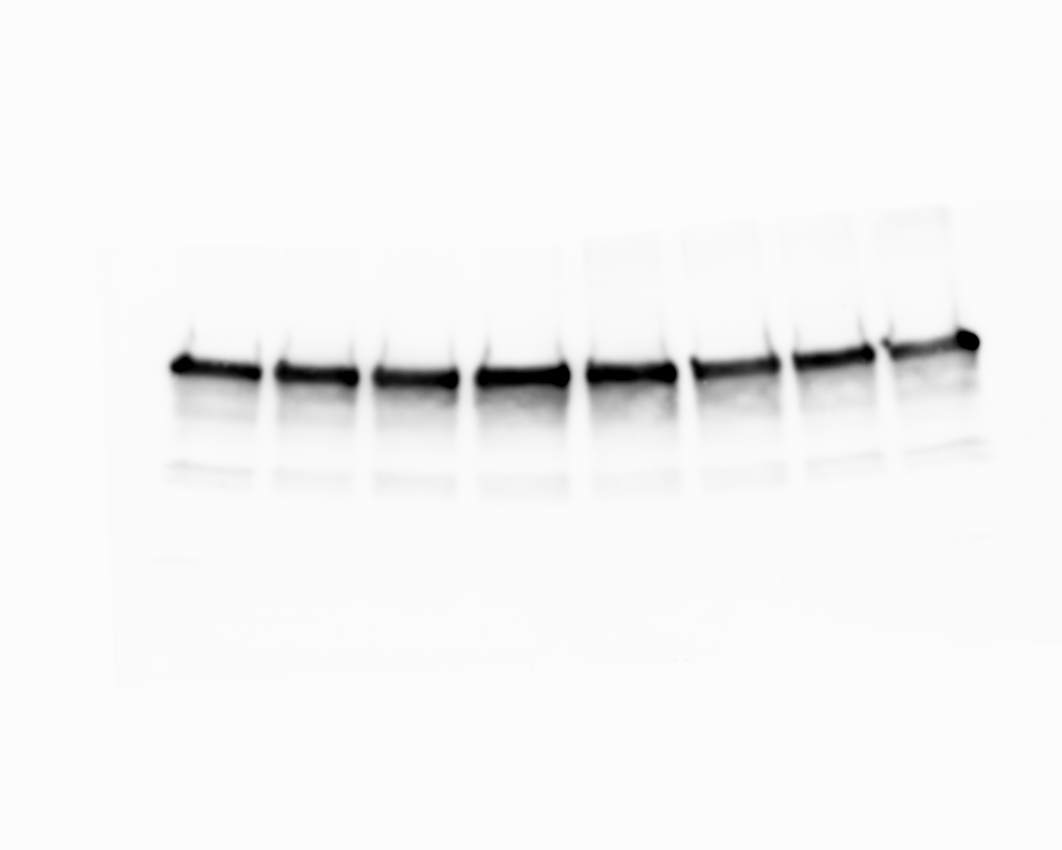

Supplement: Figure 1—source data 1. [file elife-104808-fig1-data1.zip › Raul 2025-01-11 19h59m43s(Chemiluminescence).tif]

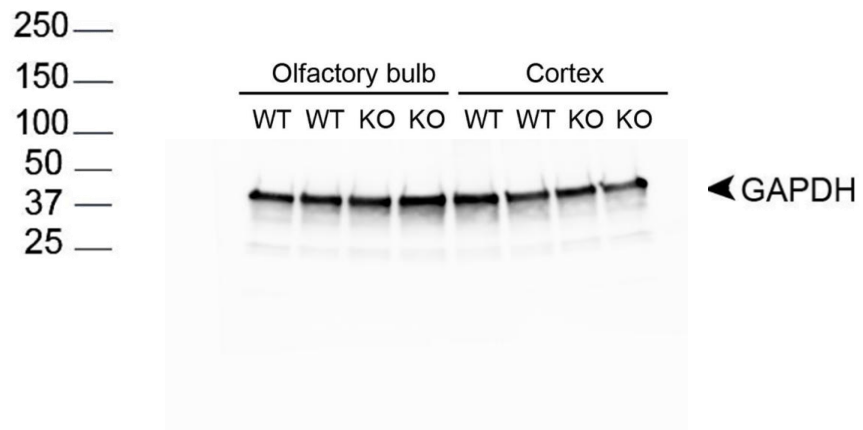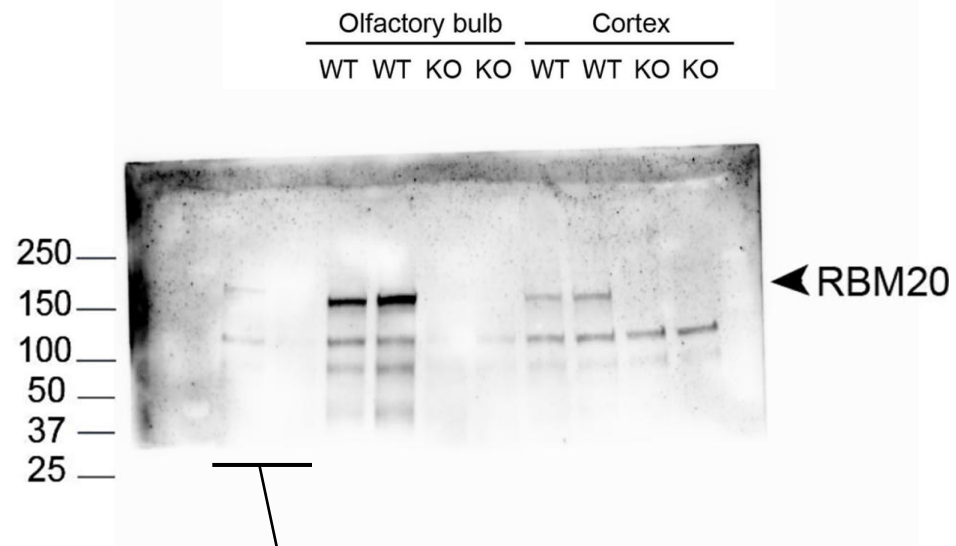

Supplement: Figure 1—source data 2. [file elife-104808-fig1-data2.zip › Figure_1_-Source_file._Labeled_Figure_File.pdf]

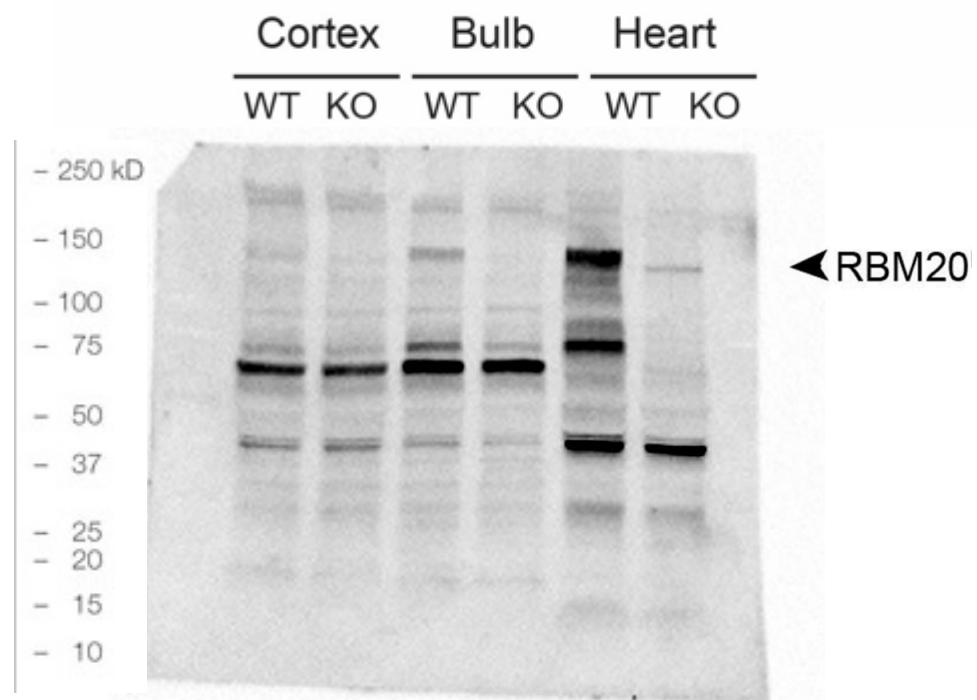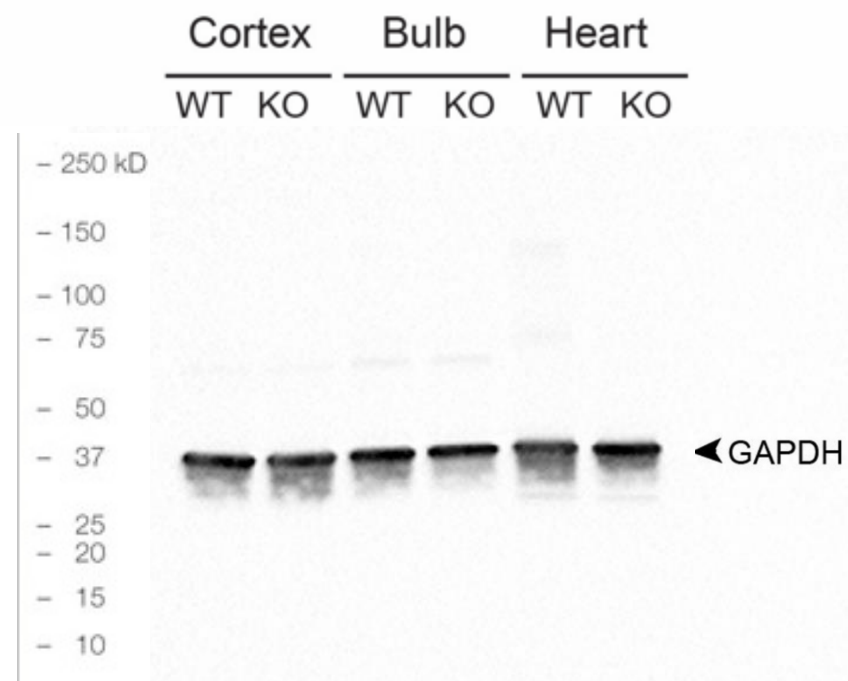

Supplement: Figure 1—figure supplement 1—source data 1. [file elife-104808-fig1-figsupp1-data1.zip › Figure S1 - Source file 1.pdf]

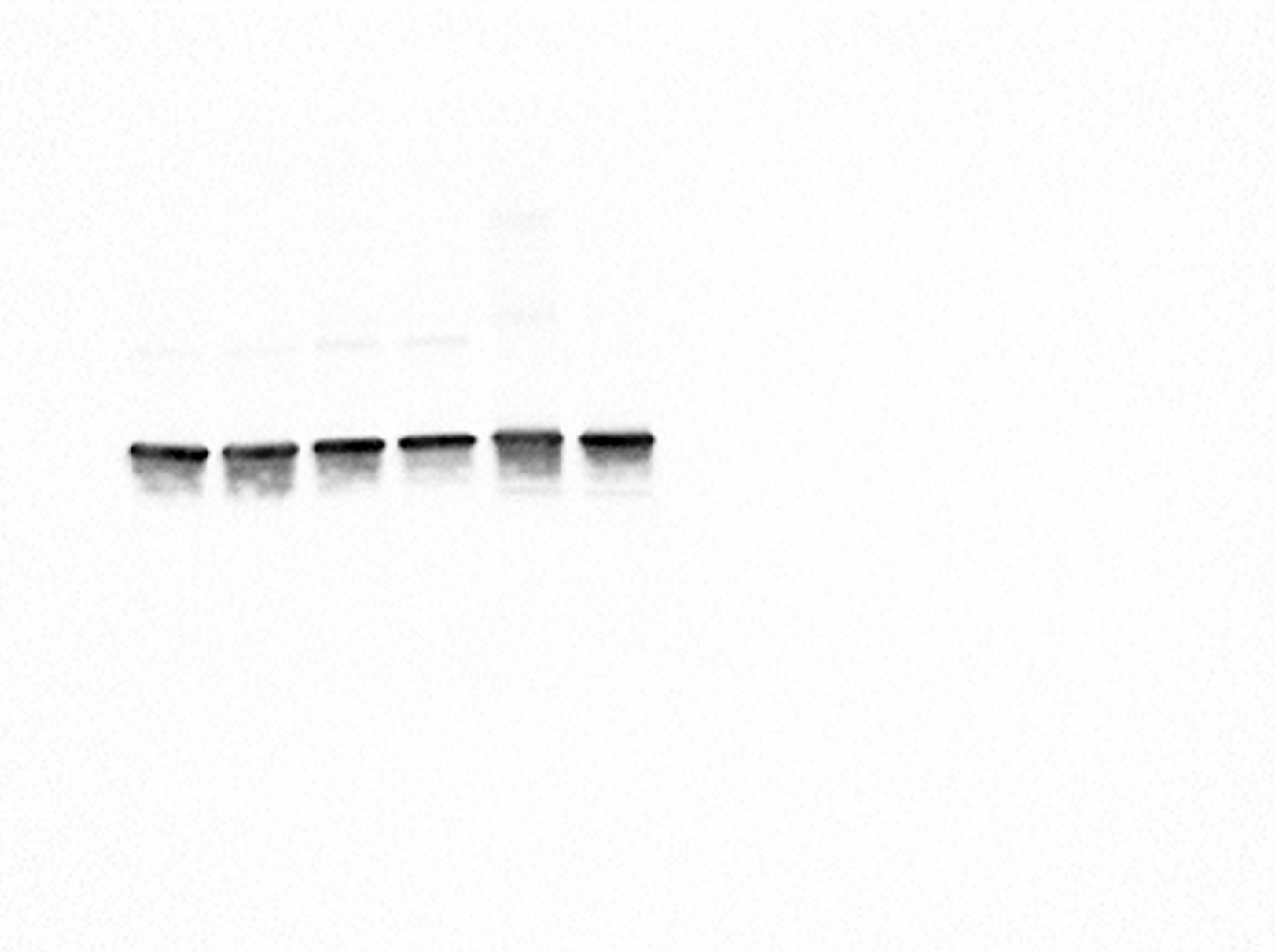

Supplement: Figure 1—figure supplement 1—source data 2. [file elife-104808-fig1-figsupp1-data2.zip › gapdh_.tif]

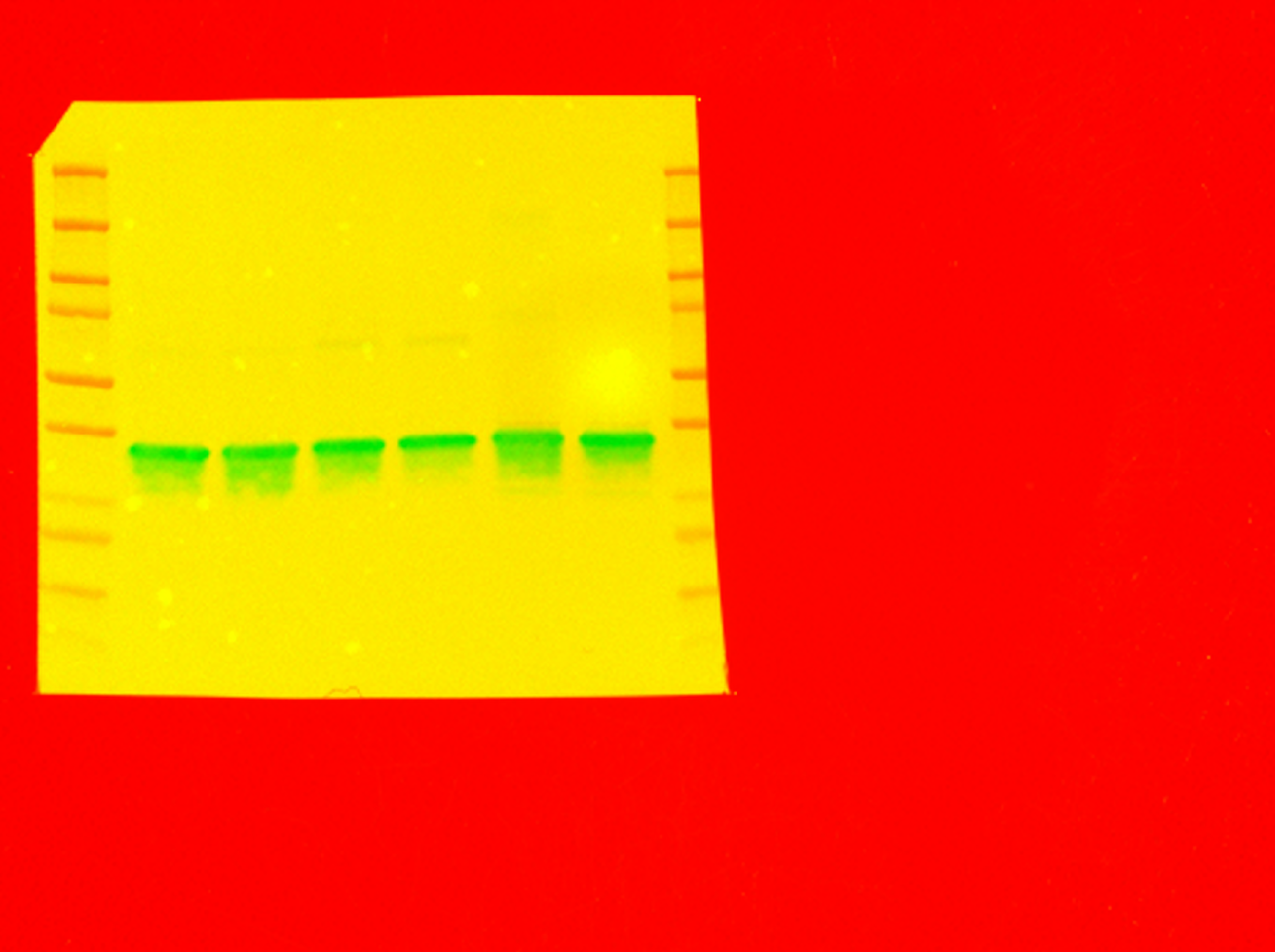

Supplement: Figure 1—figure supplement 1—source data 2. [file elife-104808-fig1-figsupp1-data2.zip › gapdh_lad.tif]

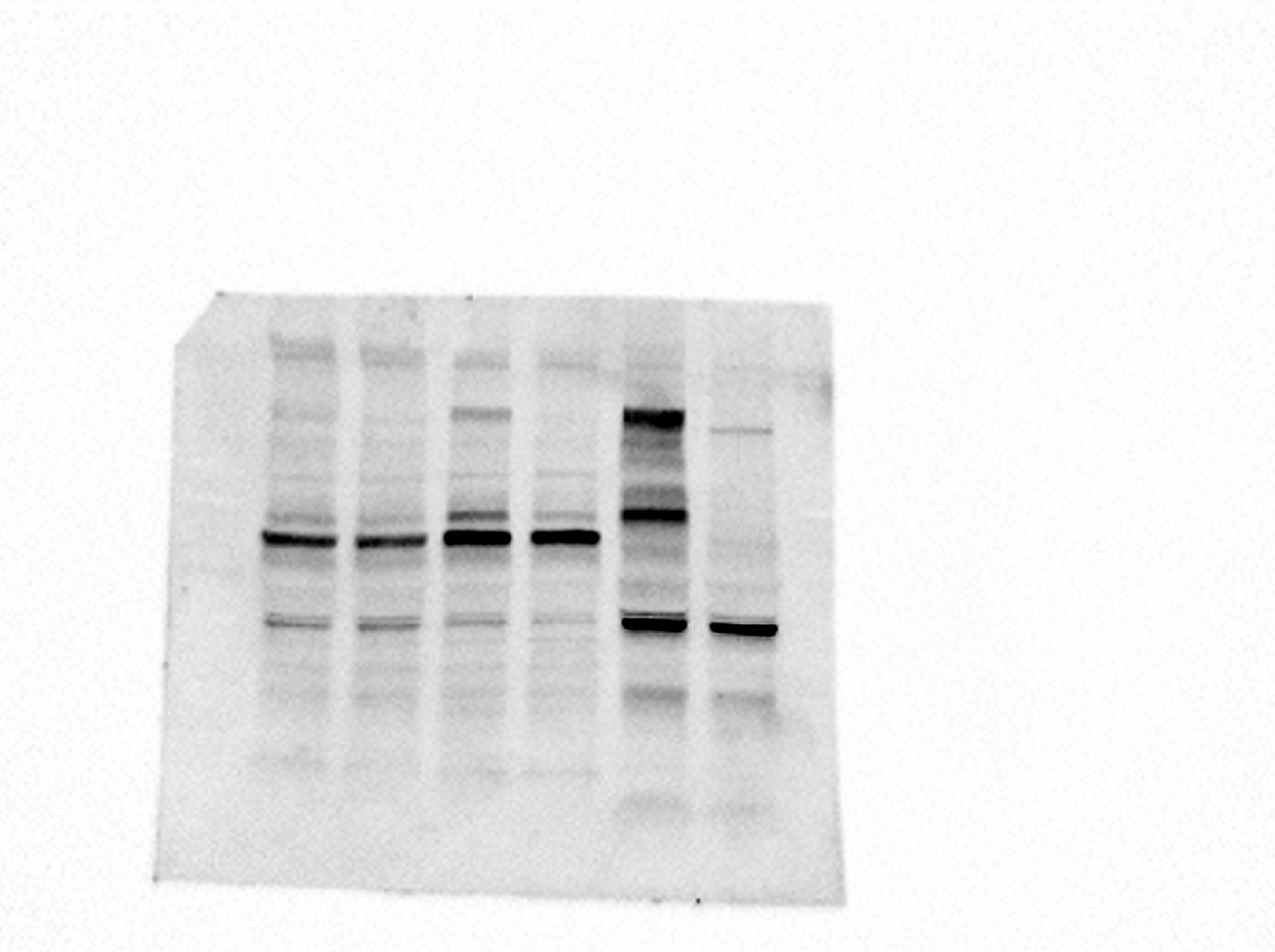

Supplement: Figure 1—figure supplement 1—source data 2. [file elife-104808-fig1-figsupp1-data2.zip › rbm20.tif]

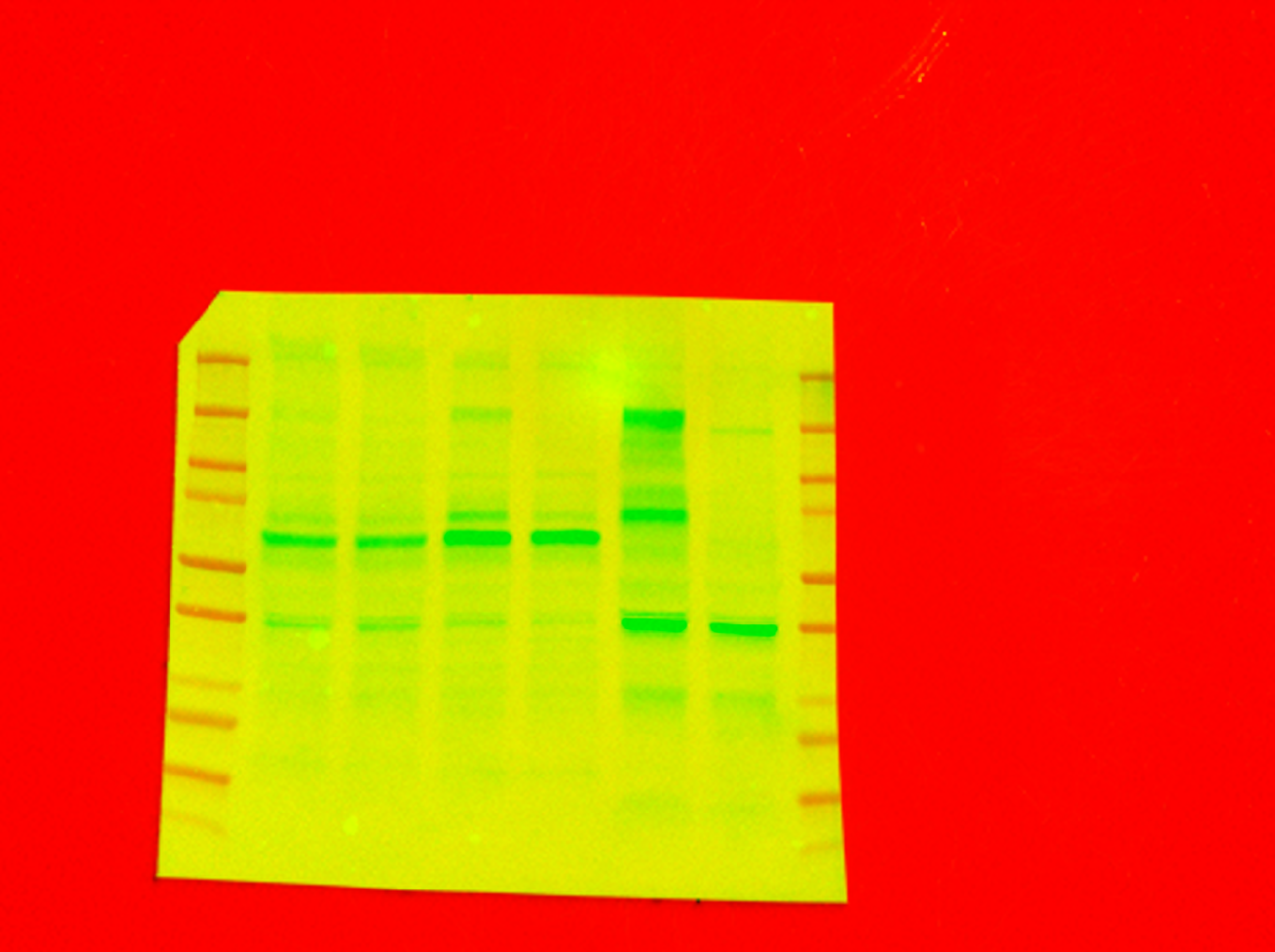

Supplement: Figure 1—figure supplement 1—source data 2. [file elife-104808-fig1-figsupp1-data2.zip › rbm20_lad.tif]

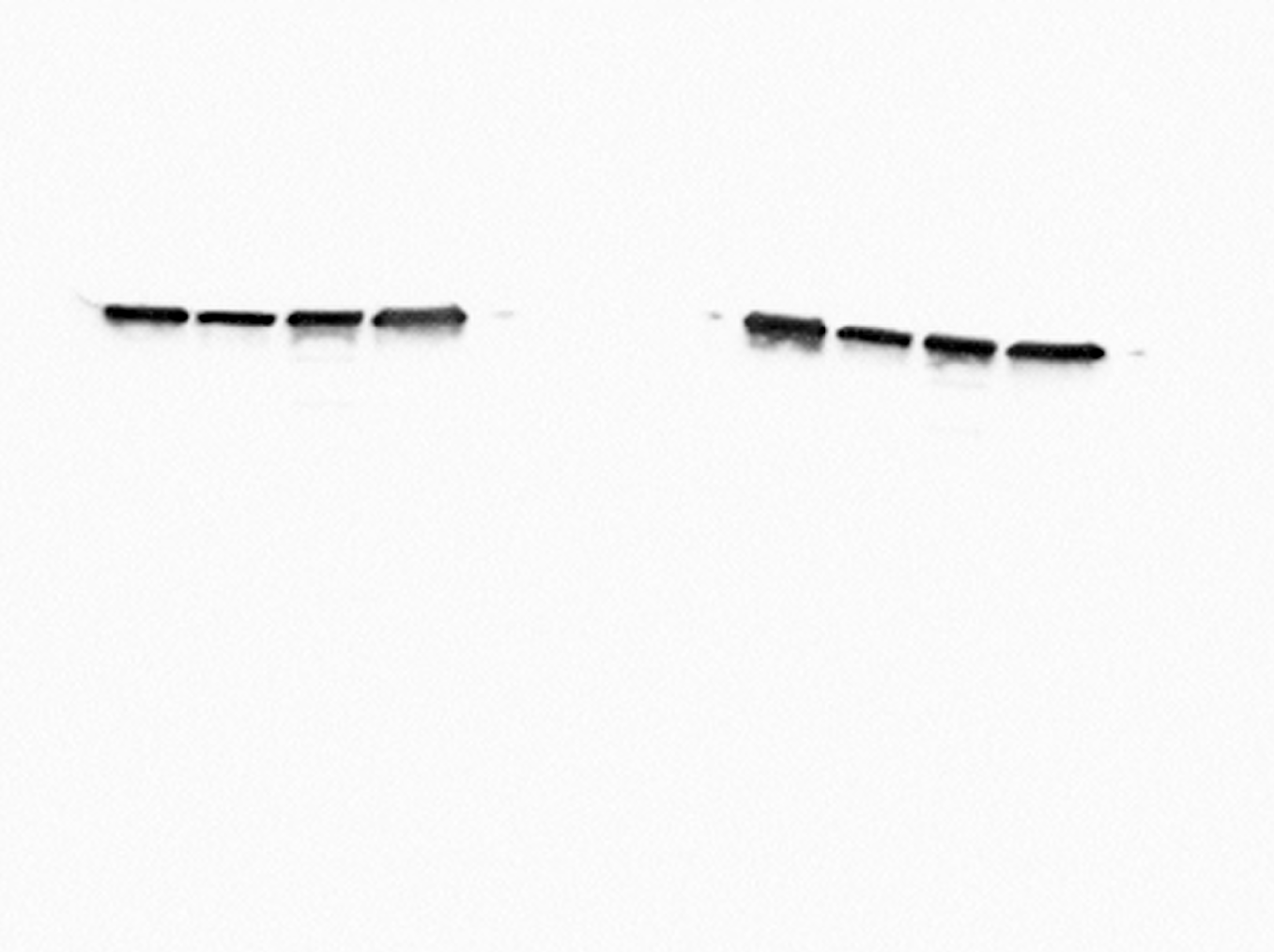

Supplement: Figure 2—source data 1. [file elife-104808-fig2-data1.zip › gapdh.tif]

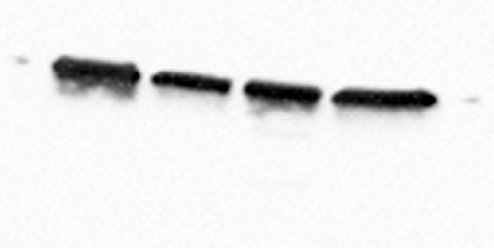

Supplement: Figure 2—source data 1. [file elife-104808-fig2-data1.zip › gapdh_cropped_photoshop.tif]

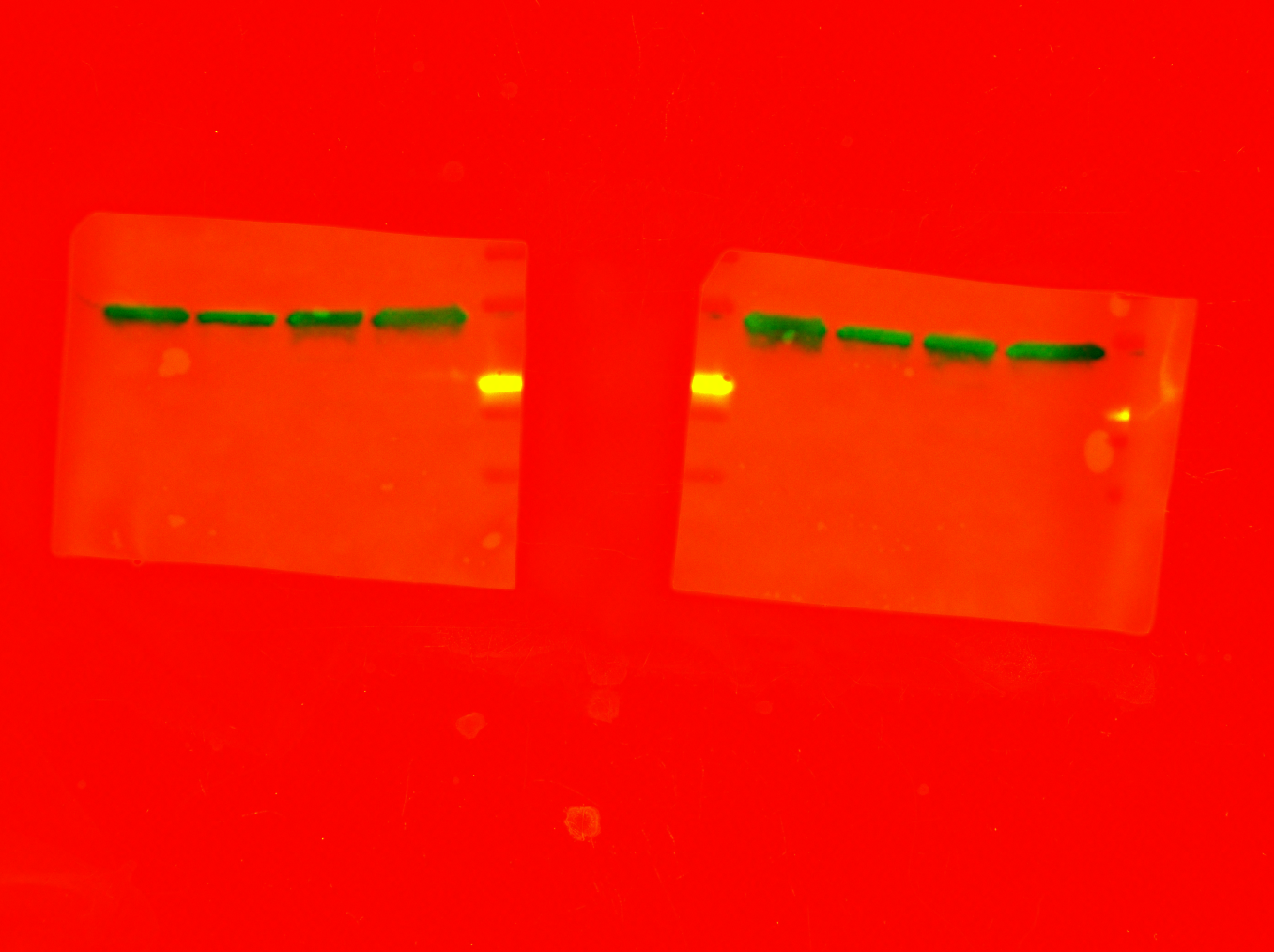

Supplement: Figure 2—source data 1. [file elife-104808-fig2-data1.zip › gapdhlad.tif]

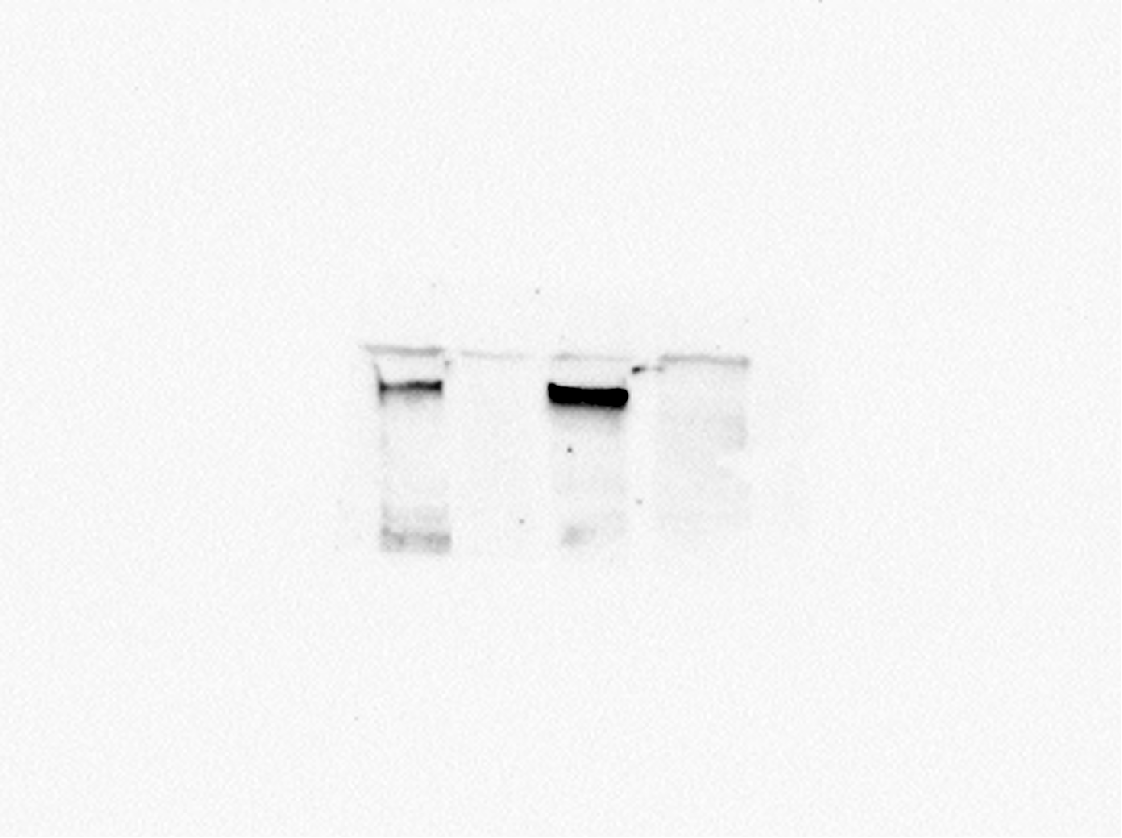

Supplement: Figure 2—source data 1. [file elife-104808-fig2-data1.zip › ha.tif]

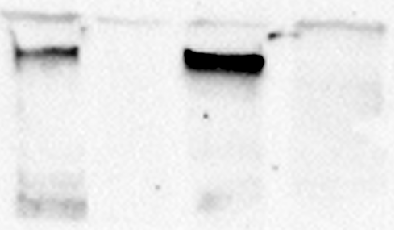

Supplement: Figure 2—source data 1. [file elife-104808-fig2-data1.zip › ha_cropped_photoshop.tif]

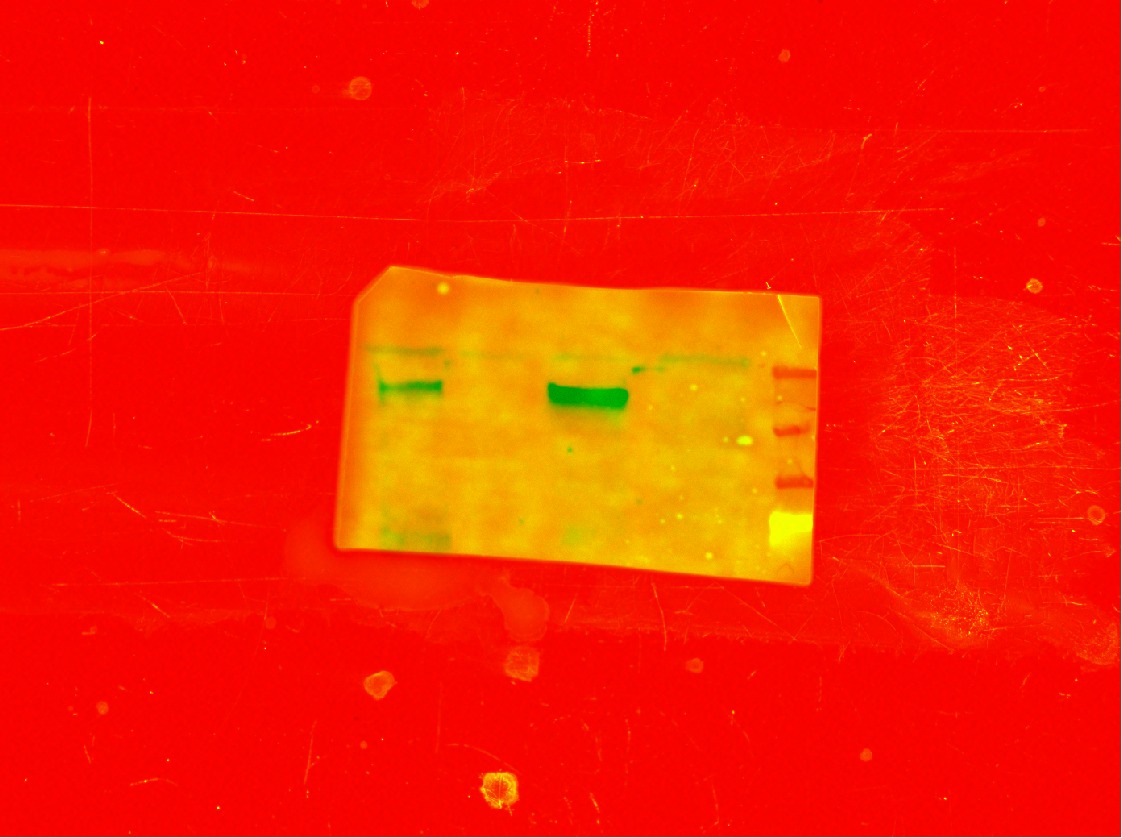

Supplement: Figure 2—source data 1. [file elife-104808-fig2-data1.zip › halad.tif]

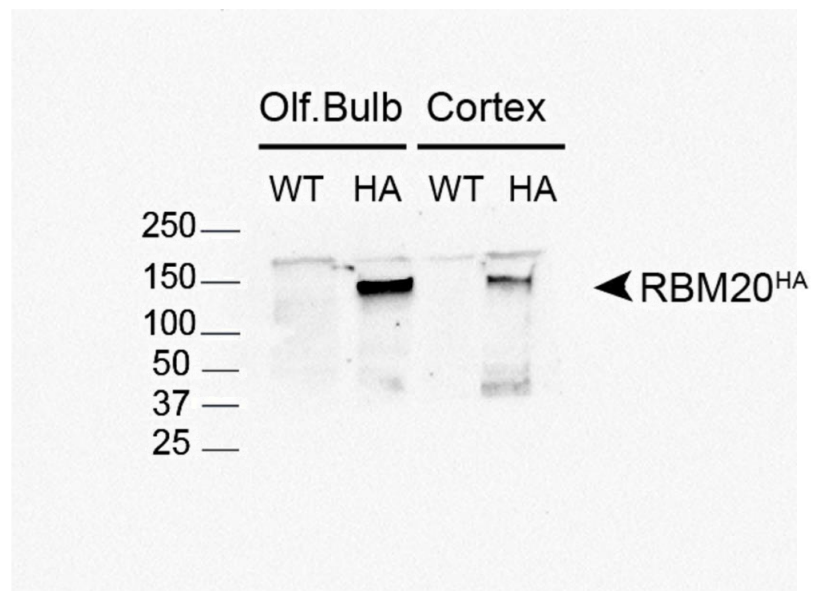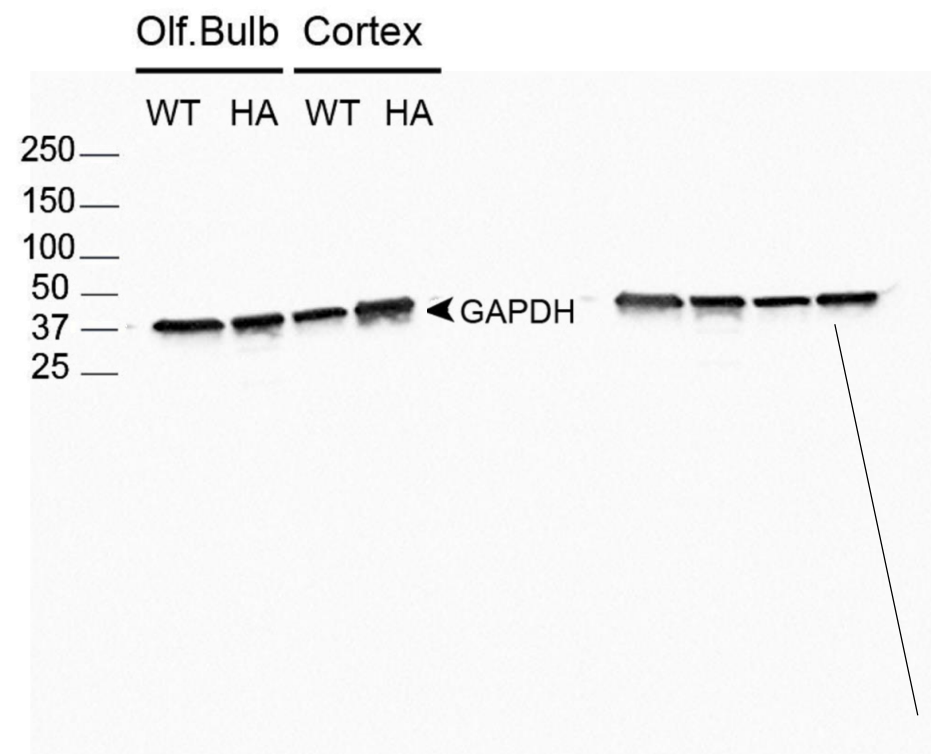

Gapdh for another blot

Supplement: Figure 2—source data 2. [file elife-104808-fig2-data2.zip › Figure 2 - Source file1.pdf]

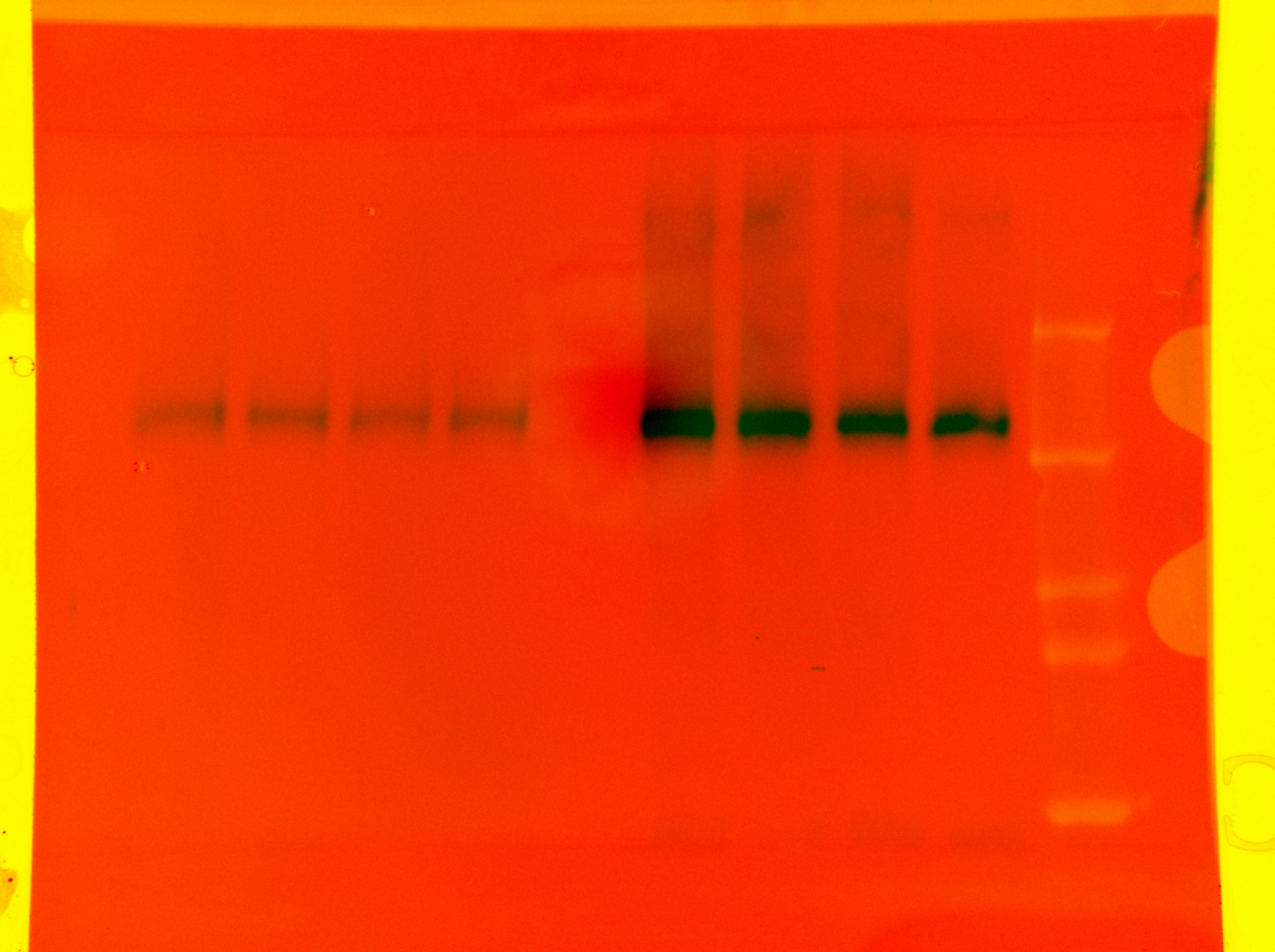

Supplement: Figure 3—figure supplement 1—source data 1. [file elife-104808-fig3-figsupp1-data1.zip › anti-HA 45 sec (Multichannel).jpg]

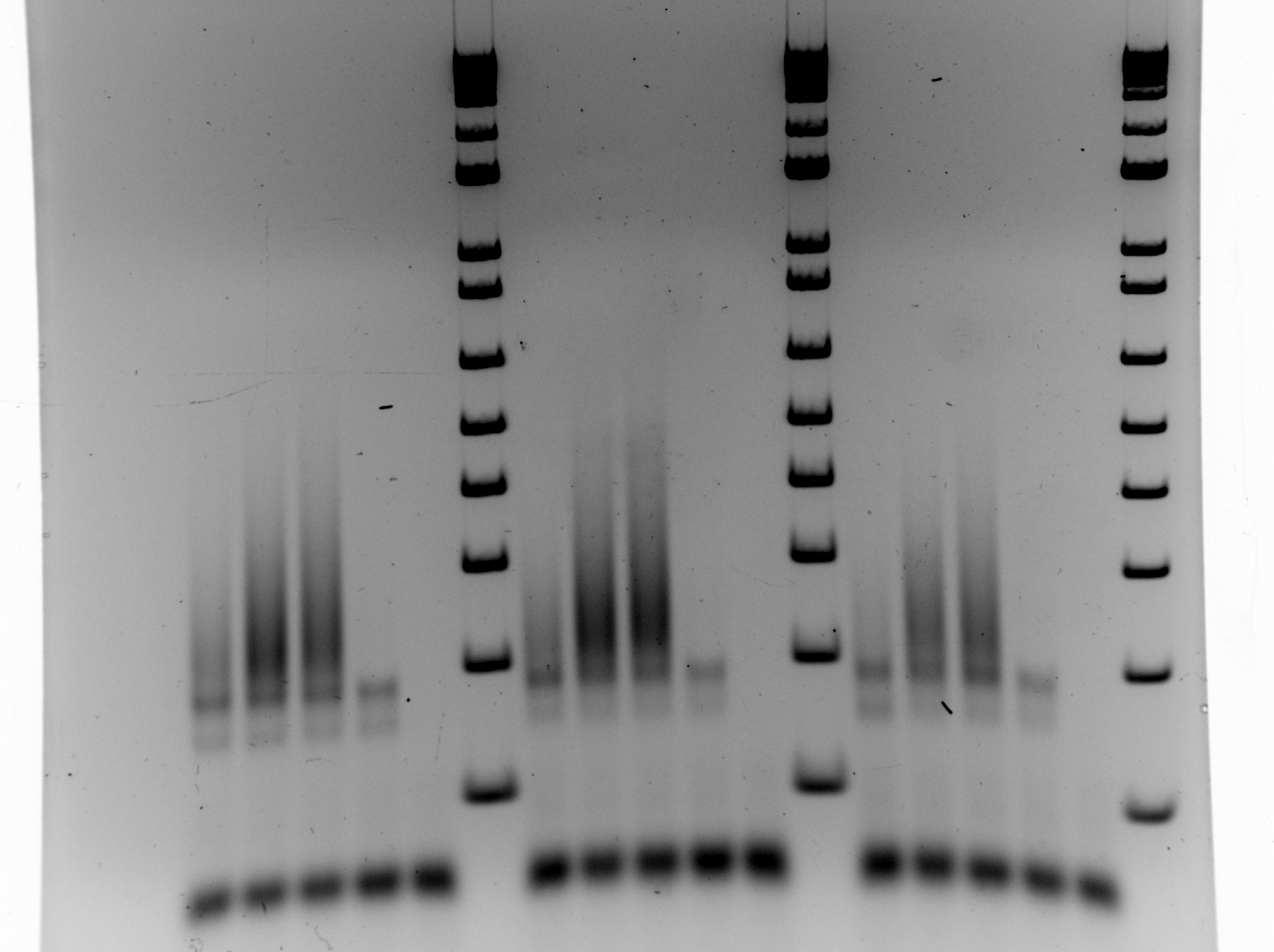

Supplement: Figure 3—figure supplement 1—source data 1. [file elife-104808-fig3-figsupp1-data1.zip › CLIP PCR 22 cycles 0.75 sec.jpg]

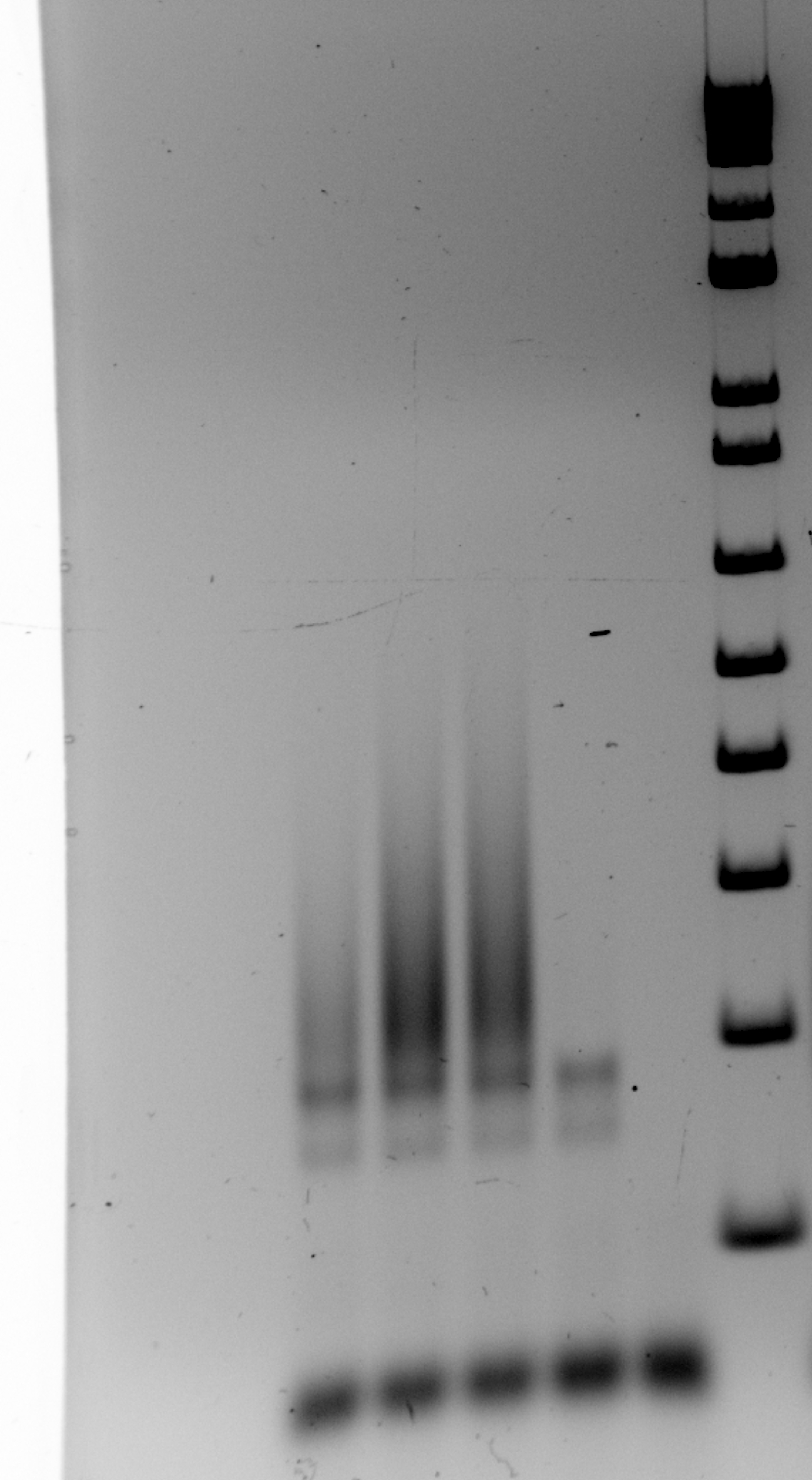

Supplement: Figure 3—figure supplement 1—source data 1. [file elife-104808-fig3-figsupp1-data1.zip › Agarose gel.tif]

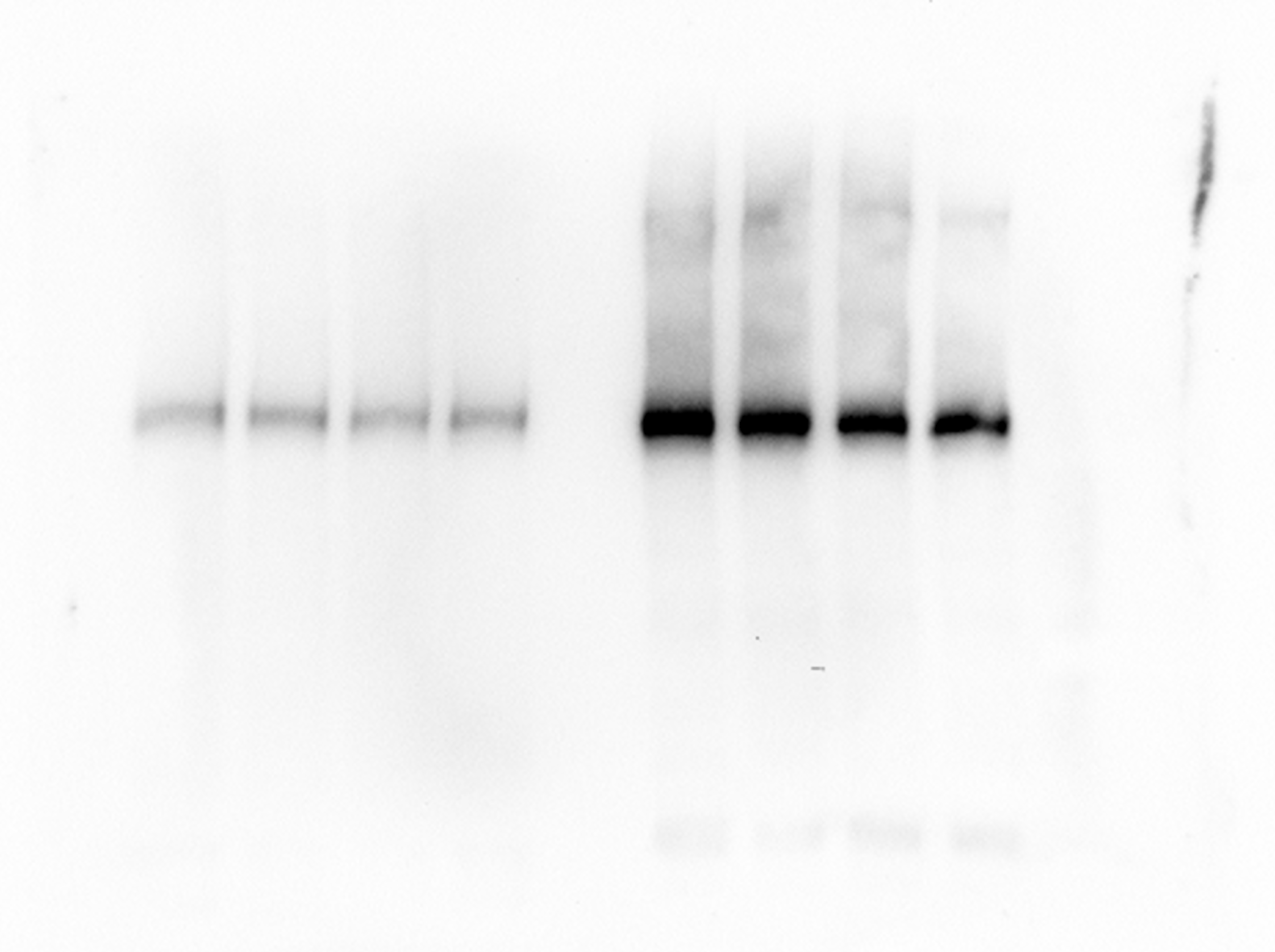

Supplement: Figure 3—figure supplement 1—source data 1. [file elife-104808-fig3-figsupp1-data1.zip › Western blot.tif]

Figure S4A

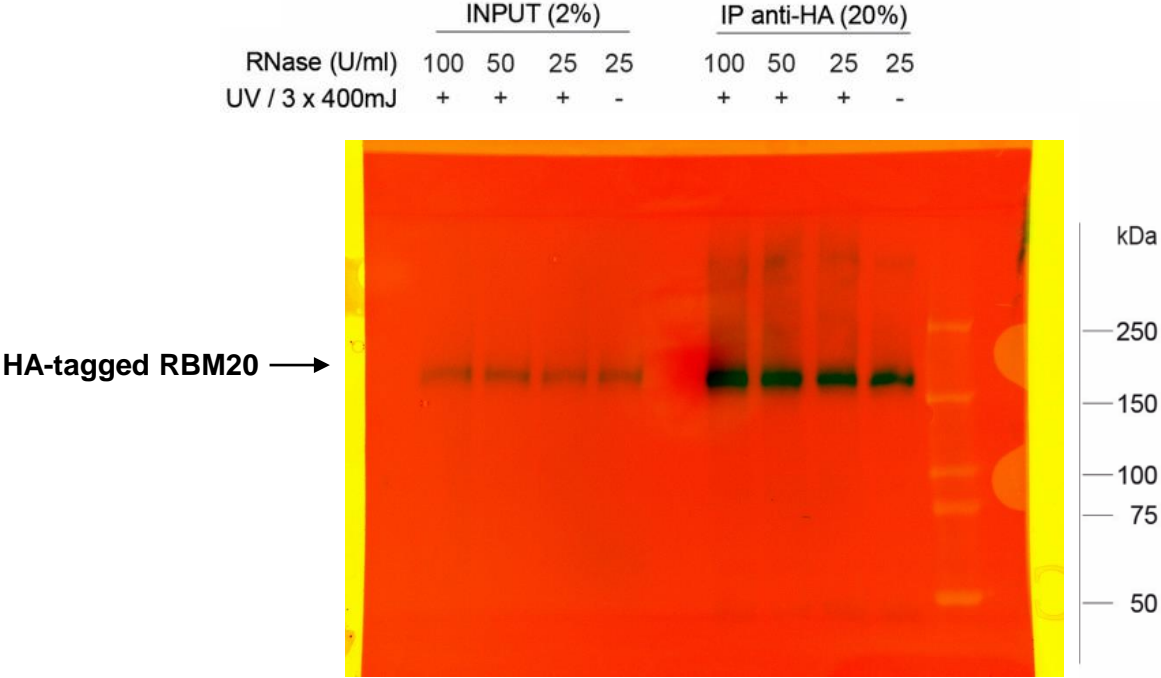

Figure S4B

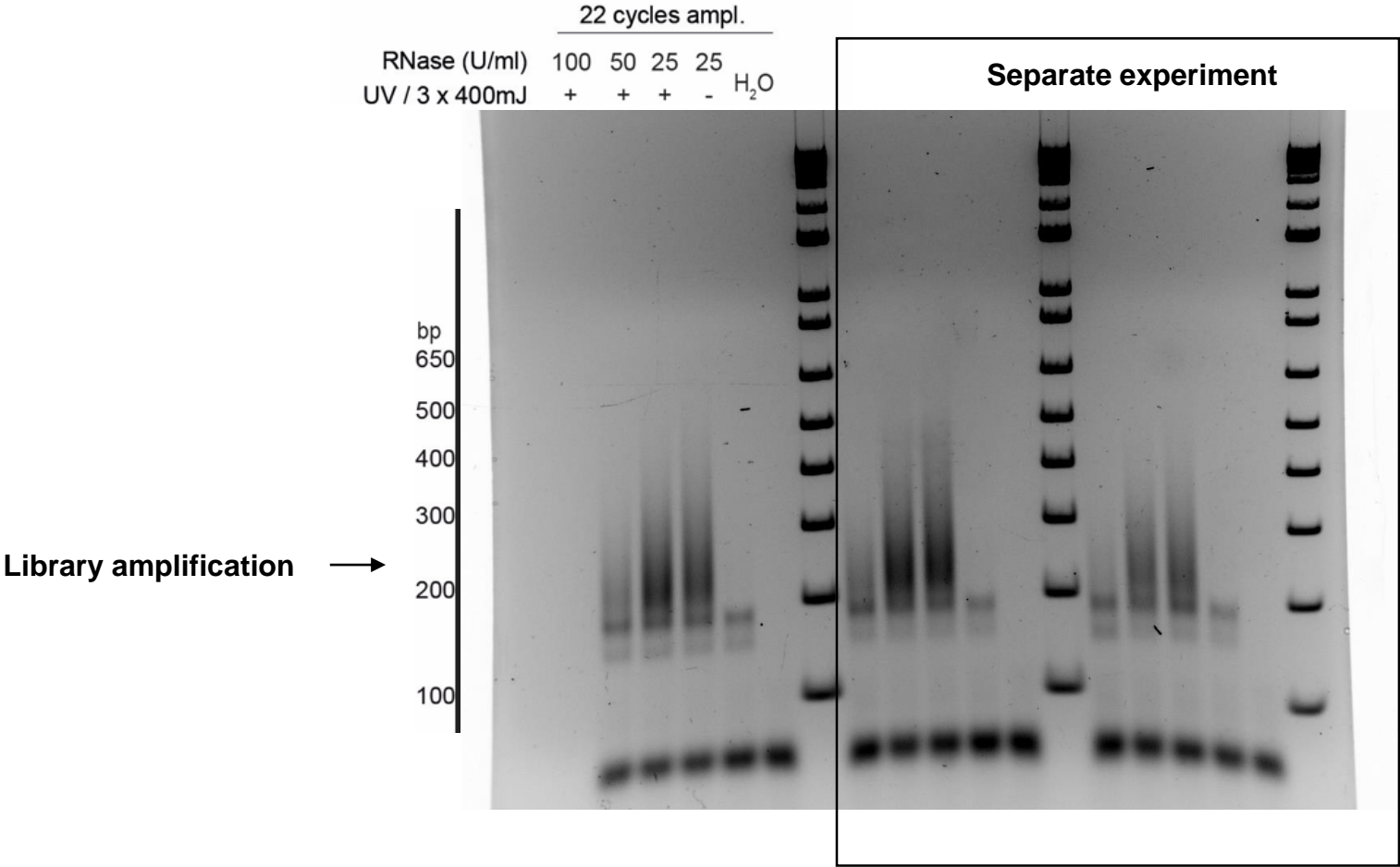

Supplement: Figure 3—figure supplement 1—source data 2. [file elife-104808-fig3-figsupp1-data2.zip › Figure_S4_-Source_file._Labeled_Figure_File-1.pdf]
